# *Chryseobacterium indologenes* in a woman with metastatic breast cancer in the United States of America: a case report

**DOI:** 10.1186/1752-1947-7-190

**Published:** 2013-07-26

**Authors:** Seema Yasmin, Greg Garcia, Tammy Sylvester, Rebecca Sunenshine

**Affiliations:** 1 Epidemic Intelligence Service, Centers for Disease Control and Prevention, 1600 Clifton Road, NE, Atlanta, GA 30333, USA; 2 Arizona Department of Health Services, 150 N 18th Avenue, Phoenix, AZ 85007, USA; 3 Maricopa County Department of Public Health, 4041 N Central Avenue, Phoenix, AZ 85012, USA; 4 Career Epidemiology Field Office, Centers for Disease Control and Prevention, 1600 Clifton Road, NE, Atlanta, GA 30333, USA

**Keywords:** Beta-lactamases, *Chryseobacterium indologenes*, *Flavobacterium*, Infection

## Abstract

**Introduction:**

We report the seventh case of *Chryseobacterium indologenes* occurring in the United States of America. *C. indologenes* is seldom isolated from clinical specimens but has caused hospital-acquired infections in Taiwan and rarely elsewhere.

**Case presentation:**

A 32-year-old Caucasian woman with metastatic breast cancer presented to a hospital emergency department with bilateral radiation-induced pleural effusions and respiratory failure. The patient was hospitalized and ventilated for 26 days; tracheal aspirates collected on ventilation days 24 and 26 grew *C. indologenes*. The patient subsequently died as a result of worsening ventilator-associated pneumonia and stage IV breast cancer.

**Conclusions:**

*C. indologenes* infection should be considered for hospitalized patients with a history of malignancy, especially those with indwelling devices and antibiotic use for >14 days.

## Introduction

We report the seventh case of *Chryseobacterium indologenes* in the United States of America (USA), occurring in a Caucasian woman aged 32 years with metastatic breast cancer. *C. indologenes* is a rare human pathogen reported to have caused hospital-acquired infections in Taiwan and rarely elsewhere: six cases have been reported in Europe, two in Australia, and four in the USA [1-4]. In addition, ophthalmic *C. indologenes* infections have been reported including one case report in the USA, one in Taiwan and one in Spain [5-7]. *C. indologenes* was first isolated from a clinical specimen in 1993 from the tracheal aspirate of a patient with ventilator-associated pneumonia. The Gram-negative bacillus is ubiquitous in soil and water, but it has not been found in human microflora and is rarely isolated from clinical specimens. *C. indologenes* can survive chlorination, is found in municipal water supplies, and can colonize sink basins and taps [8,9]. *C. indologenes* has also been found in medical devices that use water, for example humidifiers and ventilators [9]. The *Chryseobacterium* species form circular, convex, bright-yellow colonies on blood, Tryptic soy, or nutrient agar; however, it grows poorly on MacConkey medium [10]. *Chryseobacterium* bacteria are known to be resistant to extended spectrum penicillin, first and second generation cephalosporins and aztreonam; susceptibility to quinolones, aminoglycosides and cephalosporins varies. Combination antimicrobial therapy with trimethoprim-sulfamethoxazole and rifampin has been successful in treating *C. indologenes* infection [11,12]*.* Studies in Taiwan have indicated hospitalization, cancer and prolonged antibiotic treatment (>14 days) as risk factors for infection [9,13].

## Case presentation

A 32-year-old Caucasian woman was diagnosed with breast cancer and treated with radiation and mastectomy with immediate flap reconstruction and implant 1 year before the presentation of this case report. One month following the mastectomy, a pericardial effusion was identified during an out-patient thoracentesis for radiation-induced pleural effusions. The patient was admitted to a hospital where she was treated with pericardial window drainage of the effusion. She was discharged home on intravenous antibiotics (vancomycin, fluconazole) and analgesia. At home, she continued to use unspecified naturopathic therapies that she had been taking since the mastectomy and of which further details are unknown. Ten days post-discharge, the patient suffered a cardiac arrest at home and was admitted to hospital where mechanical ventilation was initiated and bilateral chest drains inserted. Eleven days later, a tracheostomy was performed. Two tracheal aspirate samples taken on ventilation days 24 and 26 grew *C. indologenes*. The isolate was susceptible to co-trimoxazole, ciprofloxacin, and levofloxacin. The patient received a diagnosis of ventilator-associated pneumonia and was treated with levofloxacin. Her white blood cell count, which had remained within normal limits until ventilation day 24, peaked at 19 × 10^9^/L on ventilation day 26. The patient died from cardio-respiratory failure and complications of stage IV metastatic breast cancer 40 days after admission.

Only five cases of non-ophthalmic and one case of ophthalmic *C. indologenes* have previously been reported in the USA. In 2001, a 77-year-old man with a history of curettage for squamous cell carcinoma of the leg developed cellulitis of the leg. Wound cultures grew *C. indologenes* that was susceptible to trimethoprim-sulfamethoxazole and levofloxacin. The patient recovered following treatment with oral levofloxacin [3].

In 2004, a case occurred in a man aged 54 years with a metastatic squamous cell carcinoma of the nasal tube. After radiation and chemotherapy, he was fed through a gastrostomy. On hospital day 46, he experienced a fever but had a normal white blood cell count. Blood cultures obtained through a peripheral venous catheter were positive for *C. indologenes,* and the patient was successfully treated with piperacillin-tazobactam and removal of the catheter [4].

A case reported in 2006 was in a Caucasian woman aged 57 years with metastatic breast cancer. An indwelling catheter through which she received chemotherapy had been colonized by *C. indologenes,* resulting in pea-sized nodules and yellow discoloration of the skin surrounding the catheter. This patient recovered after treatment with ciprofloxacin [1].

In 2007, the first case of *C. indologenes* central nervous system infection was reported in a 13-year-old boy with congenital hydrocephalus and a lumboperitoneal shunt. The patient was successfully treated with combination treatment with trimethoprim-sulfamethoxazole and rifampin [2].

The first case of subcutaneous port-related *C. indologenes* infection in a liver transplant recipient was reported in 2013 [14]. The patient, a 26-year-old woman had received a liver transplant 6 years prior to the infection and was on an established immunosuppressive regime. Blood cultures drawn from the port grew *C. indologenes*; susceptibility testing revealed resistance to piperacillin-tazobactam, other broad-spectrum β-lactams and aminoglycosides. The patient was treated with intravenous levofloxacin and oral trimethoprim-sulfamethoxazole. However, device removal was also necessary.

## Conclusions

Multiple factors placed the patient at risk for *C. indologenes* infection, including her breast cancer, recent surgery, multiple indwelling devices (for example, a peripherally inserted central catheter, bilateral chest tubes, tracheostomy, and a urinary catheter), mechanical ventilation for 39 days, and intravenous antibiotic treatment for 90 days. *C. indologenes* infection can be considered a rare opportunistic pathogen among hospitalized patients with a history of malignancy, especially those with indwelling devices and antibiotic use for >14 days.

## Consent

All reasonable efforts to obtain consent for publication from the patient's next of kin failed as they were untraceable. The authors have made every effort to ensure patient anonymity. There is no reason to believe that the patient would have objected to publication and it is not felt that anyone who knew the patient would be able to identify her from the published article.

## Competing interests

The authors declare that they have no competing interests.

## Authors’ contributions

GG, TS, RS and SY conducted the case review and contributed to the writing of the case report. All authors read and approved the final report.
